# A Rapid Process for Fabricating Gas Sensors

**DOI:** 10.3390/s140712219

**Published:** 2014-07-09

**Authors:** Chun-Ching Hsiao, Li-Siang Luo

**Affiliations:** Department of Mechanical Design Engineering, National Formosa University, No. 64, Wunhua Rd., Huwei Township, Yunlin County 632, Taiwan; E-Mail: q14703@gmail.com

**Keywords:** gas sensor, aerosol deposition, carbon monoxide, zinc oxide

## Abstract

Zinc oxide (ZnO) is a low-toxicity and environmentally-friendly material applied on devices, sensors or actuators for “green” usage. A porous ZnO film deposited by a rapid process of aerosol deposition (AD) was employed as the gas-sensitive material in a CO gas sensor to reduce both manufacturing cost and time, and to further extend the AD application for a large-scale production. The relative resistance change (ΔR/R) of the ZnO gas sensor was used for gas measurement. The fabricated ZnO gas sensors were measured with operating temperatures ranging from 110 °C to 180 °C, and CO concentrations ranging from 100 ppm to 1000 ppm. The sensitivity and the response time presented good performance at increasing operating temperatures and CO concentrations. AD was successfully for applied for making ZnO gas sensors with great potential for achieving high deposition rates at low deposition temperatures, large-scale production and low cost.

## 1. Introduction

A device capable of mimicking the olfactory discrimination mechanism is useful to many industries, not only for the detection of particular gases, but also for the quality control evaluation of mixtures in the food and beverage industries and for environmental monitoring. Gas sensors are widely used to detect and control a variety of harmful gases, thereby protecting both the atmospheric environment and human welfare. Semiconducting metal oxides have been extensively used as sensing materials; resistance changes in oxide-based semiconductor gas sensors are used to monitor reducing, toxic and inflammable gases, such as NH_3_, NO_2_, H_2_ and CO. Zinc oxide (ZnO) is a well-known functional n-type semiconductor of the II–VI group, a technologically-important, low-cost and environmentally-friendly semiconductor material, and one of the most promising candidates for detecting various gases. This semiconductor has several favorable properties, including good transparency, high electron mobility, wide bandgap, and strong room-temperature luminescence. Furthermore, ZnO is a unique material possessing such properties as semiconductivity, piezoelectricity and pyroelectricity. Because of its versatility, wide-band-gap wurtzite phase ZnO has been used in applications such as blue and ultraviolet light emitters, transparent electrodes in liquid crystal displays, solar cell windows, gas sensors, photovoltaic devices, light-emitting diodes, thin-film transistors, pyroelectric sensors, surface acoustic wave (SAW) devices and film bulk acoustic resonators (FBARs). ZnO has been extensively used as a gas-sensing material due to its high conduction electron mobility and good chemical and thermal stability when subjected to the operating conditions required in sensors. The sensing mechanism of ZnO is a surface-controlled type, in which the grain size, defects and oxygen-adsorption quantities play important roles in sensing response. It is obvious that the sensitivity and the response time of ZnO-based sensors strongly depend on their size, specific surface area and morphology [1–4]. Moreover, gas sensors based on ZnO nanobelts synthesized by RF sputtering had been investigated with a view to sensing H_2_, NO_2_ and hydrocarbon at different operating temperatures [5]. A gas sensor was fabricated from the as-prepared ZnO hollow spheres and tested to different concentrations of NH_3_ and NO_2_ at different operating temperatures [6]. The results showed that the ZnO hollow-sphere sensor exhibited extremely different sensing behaviors to NH_3_ and NO_2_. A zinc oxide nanorod-based surface acoustic wave sensor for hydrogen (H_2_) gas has been developed and investigated [7]. The ZnO nanorods were deposited onto a layered ZnO/64° YX LiNbO_3_ substrate using a liquid solution method. The study showed that the sensor responded with highest frequency shift at 265 °C. Furthermore, electronically contacted nanorods or nanotube arrays were used as gas sensors, an adsorbate modified either the impedance or the Fermi level of the array, enabling detection [8]. The arrays demonstrated the I–V curves of a Schottky diode that was formed using a metal-semiconductor junction with rectifying characteristics. The study showed that nanostructured Schottky diodes had a functionally different response, characteristic of the large electric field induced by the size scale of the array.

ZnO films have been synthesized by numerous methods, such as metal organic chemical vapor deposition, molecular beam epitaxy, magnetron sputtering, pulsed laser deposition, microwave-assisted hydrothermal processes, atomic layer deposition, spray pyrolysis, filtered cathodic vacuum arc technique, sol-gel process aerosol, and liquid-phase deposition technique [9–16]. Because nanostructures with high surface area and surface accessibility can significantly improve gas sensing properties, in this study an aerosol deposition (AD) process was used to grow porous ZnO films with a high surface-to-volume ratio for accelerating the processes and applications of the film to gas sensors. Moreover, the gas sensor was focused on the detection of carbon monoxide (CO), as CO gas is one of the most dangerous gases formed whenever incomplete combustion of carbonaceous products occurs, typically in automobile exhausts, forest fires and house fires. The AD method is based on impact adhesion of ultra-fine particles for forming and micro-pattering ceramic layers. It is a form of the gas deposition method or jet printing method without requiring vaporization of materials. In the AD, ceramic films are prepared by ejecting an aerosol mixture of ultra-fine ceramic particles and gas from the nozzle onto substrates. During the AD operation, submicron ceramic particles are accelerated by gas flow in the nozzle up to a velocity of several hundred meters per second as they are sprayed onto substrates. The AD provides many advantages for producing films in the range of 1 ∼ 100 μm thickness with a high deposition rate, low deposition temperature, large-scale production and low cost [17,18]. Moreover, the AD method can achieve fine patterning [19,20] and fabricate a dense structure through the reduction of crystallite size by fracture or plastic deformation at room temperature [17,18].

In the present study, an attempt was made to deposit a porous ZnO film with a high surface-to-volume ratio onto silicon substrates for intended use as a CO gas sensor. This was done using the AD rapid process to accelerate the deposition process of the ZnO film for its application as a gas sensor.

## 2. Experimental Procedure

### 2.1. Deposition of Porous ZnO Films

**Figure 1.**
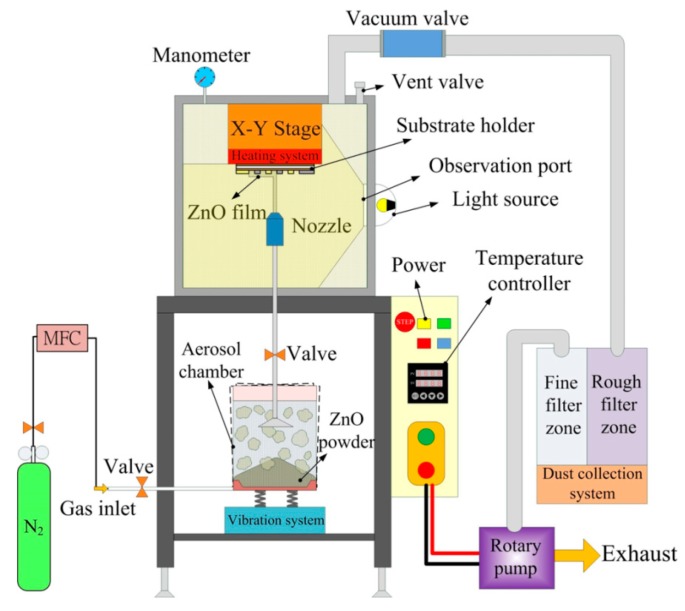
Schematic diagram of the aerosol deposition apparatus.

The AD apparatus was comprised of two vacuum chambers connected by a gas pipe. The first chamber acted as the deposition chamber for the formation of films; it included a nozzle, a motion platform and a substrate with its heating system. The chamber was evacuated during the deposition process using a rotary vacuum pump with a dust collection system. The second chamber acted as the aerosol chamber to generate ceramic aerosol; it held a carrier gas system, a filter and a vibration system for mixing the ceramic powder and the carrier gas. The ceramic aerosol in the aerosol chamber was delivered to the deposition chamber due to the pressure difference between the two chambers. The ceramic powder flowed through a nozzle and was deposited onto the substrates. The velocities of the ceramic aerosol were controlled by a mass flow controller. The schematic diagram of the AD apparatus is shown in Figure 1. Table 1 shows the deposition parameters used for the AD method.

The ZnO powder was provided by Top Nano Technology Co. Ltd., of New Taipei, Taiwan. The geometry and material properties of the starting ZnO powder are shown in Table 2. High moisture content in the ceramic powder is responsible for a reduction in film quality because agglomerated particles serve as a cushion to absorb kinetic energy when powders impact against substrates, resulting in the formation of compacted powders. Hence, a pre-treatment of the ZnO starting powder was carried out to improve the ZnO film quality by applying heat at 150 °C for 1 h using an oven. A ZnO film with a thickness of about 1 μm was deposited onto the silicon substrate by the AD at room temperature, employing various process parameters. Furthermore, the laser annealing system (LEE-25, Laser Life, Taipei, Taiwan), using continuous-wave CO_2_ laser irradiation with a wavelength of 10.6 μm, power of 25 W and beam diameter of 2 mm, was adopted for the ZnO film annealing in a N_2_ atmosphere. The laser annealing parameters comprised the movement velocity of the laser head in the X-Y plane, distance between substrates and laser head, laser power adjustment, ambient gas and flow rate, as shown in Table 3. The thickness of the ZnO films was further probed by a surface analyzer (ET-4000AK, Kosaka, Tokyo, Japan). The morphological properties were examined using scanning electron microscopy (SEM).

### 2.2. Fabrication of ZnO Gas Sensors

**Figure 2.**
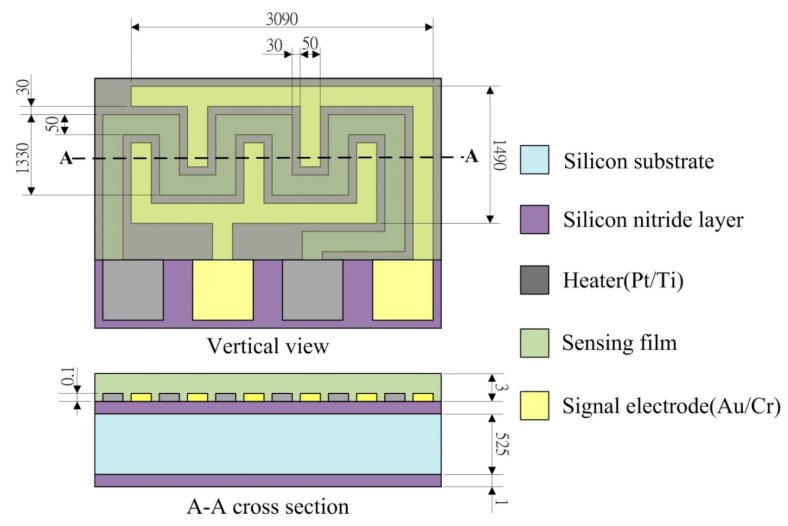
Schematic diagram of the ZnO gas sensor (unit: μm).

The ZnO gas sensor is comprised of a substrate with a silicon nitride layer, a heater (Pt/Ti), a pair of comb-like electrodes and a sensing porous ZnO film, as shown schematically in Figure 2. The width of the signal electrodes and heater was 50 μm, with a distance of 30 μm maintained between the electrodes and the heater.

**Figure 3.**
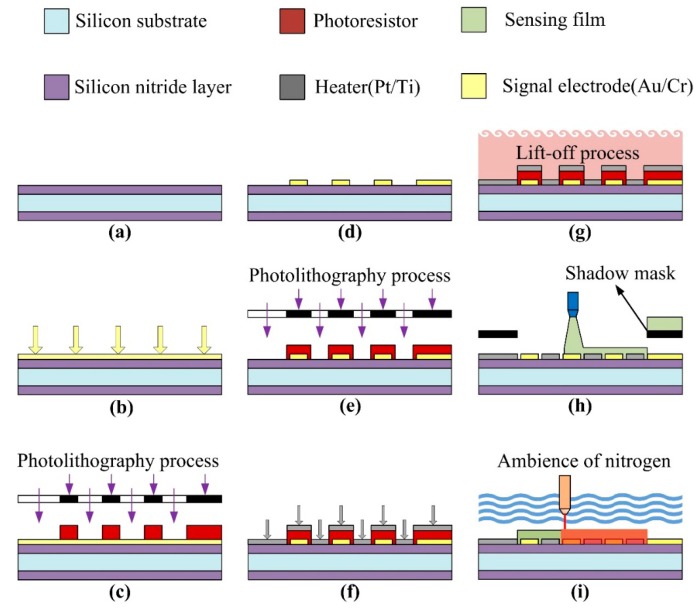
Process flow for the ZnO gas sensor.

**Figure 4.**
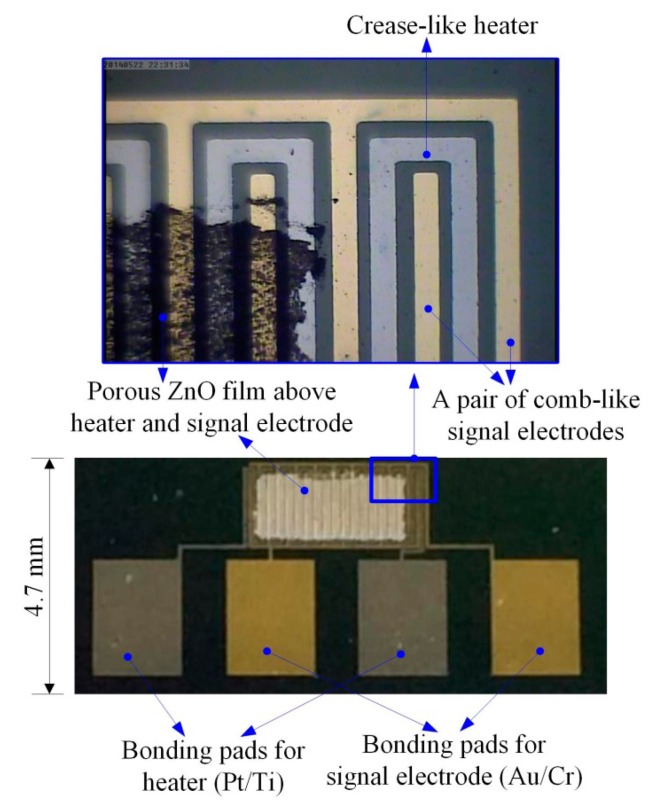
Fabricated ZnO gas sensor.

**Figure 5.**
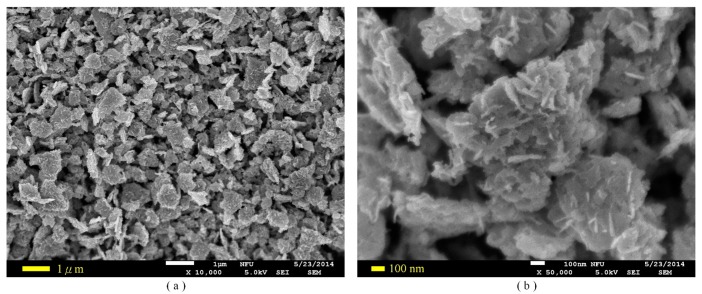
SEM images of the ZnO film deposited by AD with magnification of (**a**) 10,000× and (**b**) 50,000×.

The process flow of the ZnO gas sensor was divided into several steps, as follows. A silicon wafer with specifications of (100) p-type, double-side polished and a resistivity of 1–10 O-cm, was used as the substrate supporting the ZnO gas sensor. As shown in Figure 3a, a low-stress silicon nitride layer with a thickness of 1 μm was deposited on both sides of the substrate by LPCVD to be used as a thermal and electrical isolation layer. The pair of comb-like electrodes were deposited on the substrate by electron beam (E-beam) evaporation (as shown in Figure 3b) and patterned by photolithography (as shown in Figure 3c) with wet etching (as shown in Figure 3d). These electrodes comprised a 100 nm-thick gold layer and a 10 nm-thick chromium adhesion layer. In addition, the heater was patterned by photolithography (as shown in Figure 3e) and deposited on the substrate by electron beam (E-beam) evaporation (as shown in Figure 3f). A lift-off process was used to achieve the heater process, as shown in Figure 3g. The heater comprised a 100 nm-thick platinum layer and a 10 nm-thick titanium adhesion layer. The next step was to deposit the ZnO films about 1 μm in thickness by the AD and pattern these using the shadow mask method (as shown in Figure 3h). The ZnO film promoted by laser annealing ensued, as shown in Figure 3i. The fabricated ZnO gas sensor is shown in Figure 4. The porous ZnO architectures produced by AD were characterized by SEM, as depicted in Figure 5.

### 2.3. Gas Sensing Property Measurements

**Figure 6.**
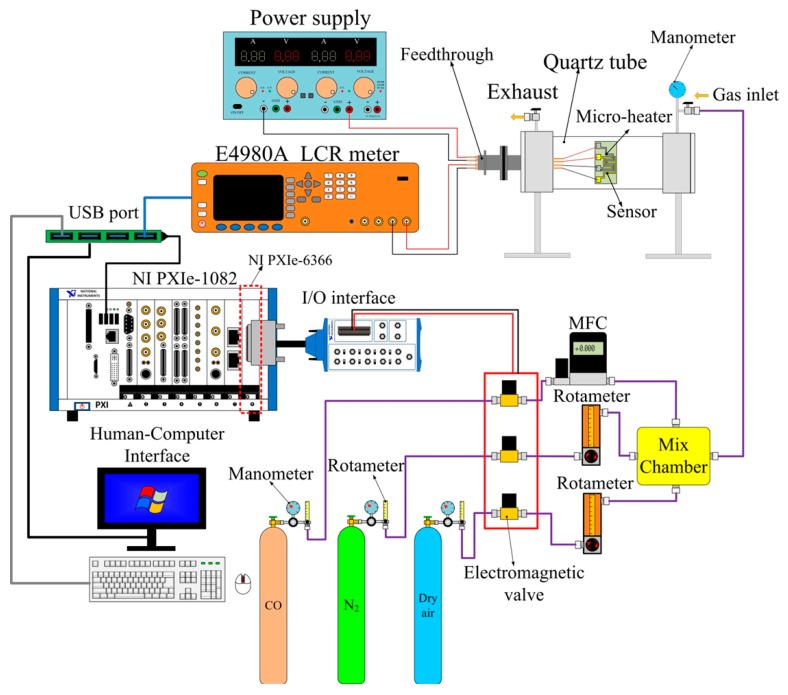
Schematic diagram of a computer-controlled gas sensing characterization system.

The electrical and CO gas-sensing properties of the fabricated gas-sensing elements were measured by a LCR meter attached to a pair of comb-like electrodes. An Agilent E4980A system formed a computer-controlled gas-sensing characterization system using a flow-through method, shown schematically in Figure 6. This automatic measurement of the gas sensing system was achieved primarily using an NI LabVIEW system consisting of a NI PXIe-1082 case, a NI PXIe-8135 controller, a NI PXIe-6366 data acquisition card and NI LabVIEW 2012 software. The gas sensors were placed in a sealed quartz tube acting as an airtight chamber possessing an inlet, an outlet and electrical feed-through. Carrier gas (dry air) and target gas (CO with N_2_) were loaded into the sealed chamber through the inlet port while the gases automatically exited the outlet port due to the difference in pressure between the interior and exterior of the chamber. During the whole measurement, dry air was continuously introduced into the chamber at a flow rate of 500 sccm. The target gas (CO) at an initial concentration of 5000 ppm with nitrogen balance and coordination was further diluted in dry air (flow rate of 500 sccm) for controlling the CO gas concentration. The operating temperature was controlled using a micro-heater consisting of platinum and titanium layers integrated within the ZnO gas sensor.

## 3. Results and Discussion

**Figure 7.**
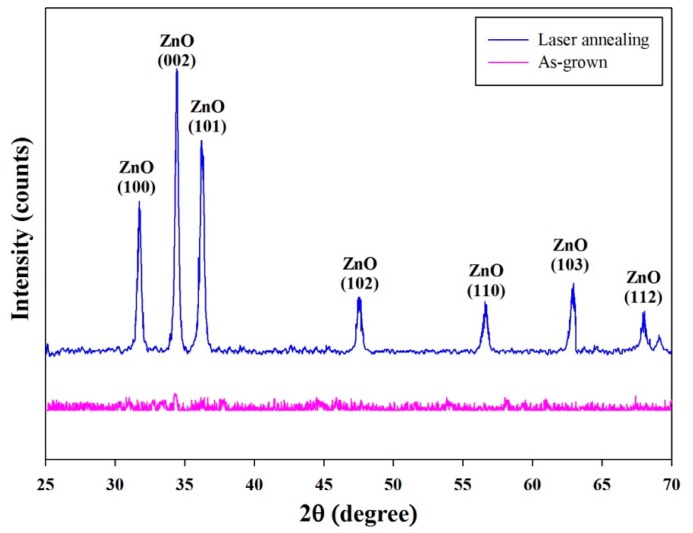
The XRD pattern of the aerosol deposited ZnO film.

The phase and structural analysis of the aerosol deposited ZnO films was carried out by XRD and is shown in Figure 7. The XRD pattern exhibited typical peaks at 2θ = 31.80°, 34.60°, 36.20°, 47.50°, 56.60°, 62.90° and 67.90° belonging to the reflection of (100), (002), (101), (102), (110), (103) and (112) of the wurtzite hexagonal ZnO, respectively, with the lattice constants of a = 0.325 nm and c = 0.521 nm (JCPDS card, No. 36-1451). The X-ray diffractogram indicated the growth of the ZnO film treated by laser annealing along the seven planes of orientations (100), (002), (101), (102), (110), (103) and (112). Though prominent growth occurred along (100), (002) and (101), the most preferential growth was observed along the (002) plane with the FWHM of 0.26°.

The CO gas-sensing properties of the porous ZnO film gas sensors were measured by using various CO gas concentrations. The gas-sensing mechanism of the semiconducting metal oxide gas sensors is based on variations in electrical resistance or conductance due to gas adsorption and desorption on the sensor surface. When the ZnO gas sensors are exposed to air, oxygen molecules from the ambient air adsorb on the exposed ZnO surface to form chemisorbed oxygen anions; (O_2_^−^, O^−^ or O^2−^, depending on the operating temperature) by capturing electrons from the ZnO conduction band. This results in the formation of a depletion layer, known as the space charge layer, on the surface of the ZnO sensing material. This induces an increase in the resistance of the sensing materials. The reactions under various operating temperatures can be described as follows:

(1) $${\text{O}}_{2}+{\text{e}}^{-}\to {\text{O}}_{2}^{-}$$

(2) $${\text{O}}_{2}+2{\text{e}}^{-}\to 2{\text{O}}^{-}$$

(3) $${\text{O}}_{2}+4{\text{e}}^{-}\to 2{\text{O}}^{2-}$$

When the ZnO gas sensors are exposed in a CO environment at a moderate temperature, the adsorbed CO gas reacts with the chemisorbed oxygen anions on the ZnO surface, resulting in CO_2_ molecules and the release of electrons back into the conduction band. This leads to an increase in the concentration of electrons. The resistivity of the ZnO films decreases for detecting the CO gas. The reactions under various operating temperatures can be described as follows:

(4) $$2\text{CO}+{\text{O}}_{2}^{-}\to 2{\text{CO}}_{2}+{\text{e}}^{-}$$

(5) $$\text{CO}+{\text{O}}^{-}\to {\text{CO}}_{2}+{\text{e}}^{-}$$

(6) $$\text{CO}+{\text{O}}^{2-}\to {\text{CO}}_{2}+2{\text{e}}^{-}$$

**Figure 8.**
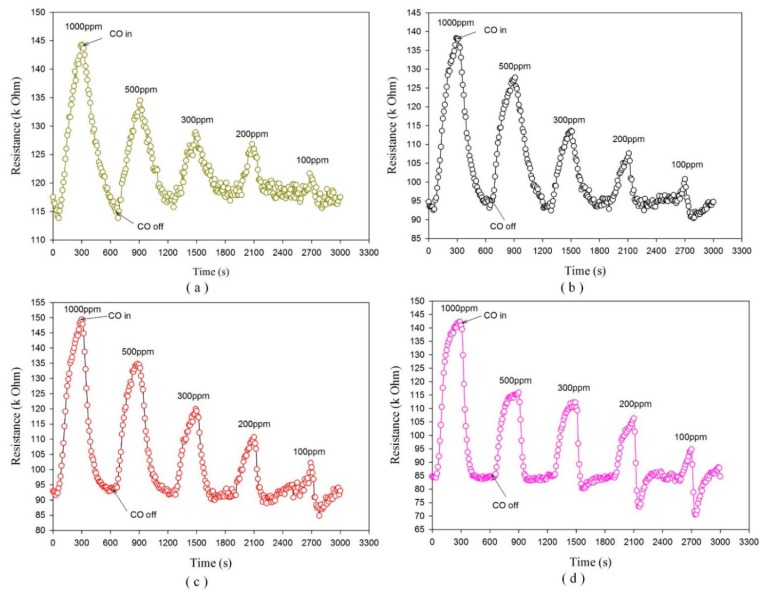
Variation in resistance of ZnO gas sensors during the cycles of CO injection and termination under various operating temperatures, (**a**) 110 °C; (**b**) 140 °C; (**c**) 160 °C; (**d**) 180 °C.

**Figure 9.**
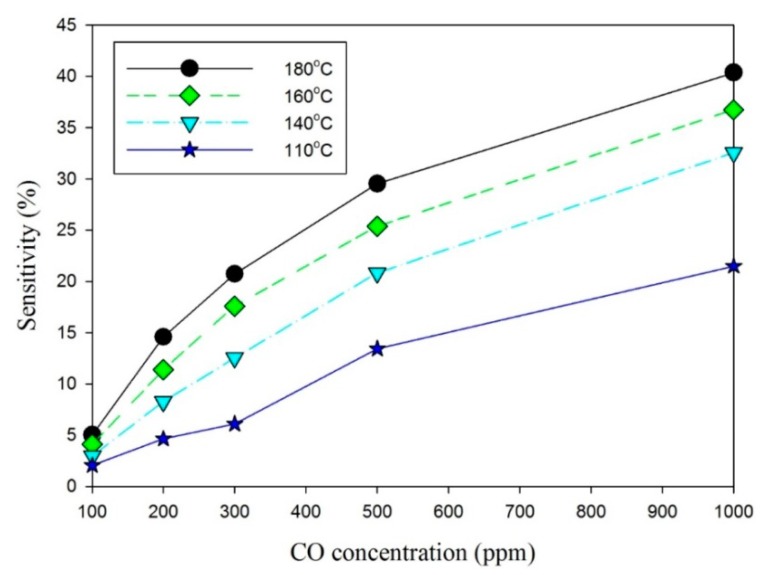
Relationship between CO concentrations and sensitivity of the gas sensors under various operating temperatures.

Because ZnO is a low-conductive semiconductor material, oxygen ion sorption and the transfer of electrons is not possible at room temperature. The dynamic resistance transients with various tested temperatures and CO concentrations are depicted in Figure 8. The initial resistance of the ZnO gas sensor in dry air was high, whereas its resistance decreased abruptly when it was exposed to CO gas. The resistance of the ZnO gas sensor decreased after each CO gas injection cycle. This exhibits n-type semiconducting behavior of the ZnO material, and its response and recovery process can be established by comparing the resistance of the ZnO nano-architectures in different ambiences. CO is a well-known reducing gas that can provide electrons to n-type ZnO semiconducting nano-architectures. The resistance variation between the injection cycles of CO and dry air increased with increasing test temperatures and CO concentrations. Moreover, the lower initial resistance at higher temperatures was also ascribed to the semiconducting property of the ZnO material. It was clearly seen that the response characteristics of the sensor depended on the operating temperature. The sensitivity (S_CO_) of the porous ZnO gas sensors is defined as a ratio of the change in resistance between samples exposed in dry air and in CO gas:

(7) $${\text{S}}_{\text{CO}}=({\text{R}}_{\text{a}}-{\text{R}}_{\text{CO}})/{\text{R}}_{\text{a}}$$

where R_a_ is the resistance of the samples measured in air, and R_CO_ is the resistance of the samples measured in CO gas. The resistance is measured between the pair of comb-like electrodes via the ZnO film deposited by the AD method. Figure 9 shows the relationship between CO concentrations and sensitivity observed for the gas sensors at various operating temperatures. The sensitivity of the sensors increased as CO concentrations and operating temperatures increased. It is well known that the sensitivity of the sensor based on sensing oxides was mainly determined by the interactions between the target gas and the sensing surface. Furthermore, it can ensure that the surface area of the sensing materials is greater, and the stronger interaction and the higher response can be expected [4]. The ZnO nanoarchitectures fabricated by the AD are shown in Figure 5; with interlaced-connected network, they maintained the high surface area and efficiently avoided the agglomerated configuration, resulting in an excellent response. This architecture presented a stacked structure with ZnO nanosheets. Moreover, some nanoprotrusions were attached onto the ZnO nanosheets, and they were produced by laser annealing to increase the sensing surface.

**Figure 10.**
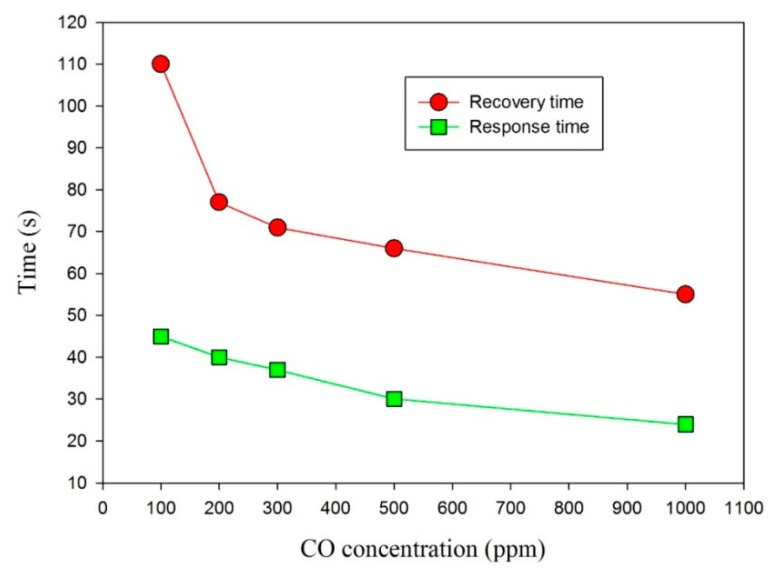
Relationship between CO concentrations, and the response and the recovery time for the gas sensors at a higher operating temperature of 180 °C.

The response and recovery time are important characteristics for appraising gas sensors, and they determined the surface accessibility of the sensing framework. Furthermore, the response time included the gas diffusion toward the sensing surface for reacting with chemisorbed oxygen ions, and the subsequent re-oxidation process of the sensing surface to yield oxygen species. The response and recovery time were defined as the time required for the gas sensor to achieve 90% of the total resistance change in the case adsorption and desorption, respectively. The response and the recovery time were related to operating temperatures; they shrank to increase the operating temperature. Figure 10 shows the relationship between CO concentrations, and the response and recovery time for the gas sensors at a higher operating temperature of 180 °C. The response time was smaller than the recovery time at each CO concentration. This was effective for practical application because the gas sensors generally readied in an ambiance of dry air. Therefore, a short response time was useful for detecting the variation of CO concentrations. The response time ranged from 24 s to 45 s, whereas the recovery time ranged from 55 s to 110 s under CO concentrations varying from 1000 ppm to 100 ppm. Furthermore, the ZnO gas sensor for CO gas-sensing properties was estimated by a circulation of CO injection about 5 min and an air ambiance about 5 min. The resistance curve was like to a triangle shape while the ZnO gas sensor worked at the lower operating temperature. This meant that the total resistance change of the ZnO film needed more time. Although the response and recovery time for observing the total resistance change of the ZnO film increased at the lower operating temperature, the sensitivity of the ZnO gas sensor at the lower operating temperature was still ponderable.

The possible model for clarifying the gas-sensing mechanism of the nanorods' film gas sensors had been demonstrated [21]. The three types of junctions: point-junction, cross-junction and block-junction formed the potential barriers and blocked electrons percolating in ZnO nanorods network. Moreover, the potential barrier of the point-junction formed by the two depletion layers of the two contact nanorods was more significantly modified upon exposure to the target gas compared with that of the block and the cross-junction. In the present study, the ZnO nanoarchitecture deposited by the AD was a porous ZnO film with a stacked structure. The randomly-stacked ZnO film possessed a larger surface area for reacting to CO gas and absorbing more CO gas. Furthermore, the proposed ZnO film presented more junctions between nanosheets for enhancing the resistance variation and increasing the surface accessibility. Therefore, the ZnO film grown by the AD using pure ZnO powder results in a low-cost, rapid, convenient, large-scale and simple process for fabricating gas-sensing devices.

## 4. Conclusions

The rapid AD process is proposed for application to gas sensors. This may minimize production costs, reduce the necessity of additional post-assembly processes and diminish the scope of fabrication processes. Zinc oxide (ZnO) is an environmentally-friendly material, which can be used in producing green products and devices. The AD process was successfully used to accelerate the application of ZnO films onto CO gas-sensing devices. The resulting CO gas sensor demonstrated improved performance, yet it can still be fabricated more quickly and at a lower cost.

## Figures and Tables

**Table 1.** Deposition parameters for AD.

Item	Data
Starting powder	ZnO (530 nm in average diameter)
Pressure difference between deposition and aerosol chambers	140 (Torr)
Carrier gas	Nitrogen
Consumption of carrier gas	3 (L/min)
Orifice size of nozzle	0.4 × 10 (mm × mm)
Substrate temperature	25 (°C)
Deposition area	70 × 70 (mm × mm)
Distance between nozzle and substrate	5 (mm)
Scanning rate	10 (mm/s)
Deposition rate	8.2 (nm/s)

**Table 2.** Geometry and material properties of ZnO powder.

Item	Data
Appearance	White powder
Density	5.61 g/cm^3^
Specific surface area	17 m^2^/g
Particle form	Sheet
Particle size	300 nm (Diameter) × 20 nm (Thickness)

**Table 3.** Process parameters for ZnO film treated by laser annealing.

Item	Data
Laser type	Continuous-wave CO_2_ laser
Laser power	25 (W)
Movement velocity of laser head	1.4 (mm/s)
Distance between substrates and laser head	40 (mm)
Ambient gas	Nitrogen
Flow rate of ambient gas	3 (L/min)
Laser spot	2 mm (diameter)
